# Accelerating implementation of research in Learning Health Systems: Lessons learned from VA Health Services Research and NCATS Clinical Science Translation Award programs

**DOI:** 10.1017/cts.2020.25

**Published:** 2020-03-17

**Authors:** Amy M. Kilbourne, Patricia L. Jones, David Atkins

**Affiliations:** 1 Health Services Research and Development, Veterans Health Administration, U.S. Department of Veterans Affairs, Washington, DC, USA; 2 Department of Learning Health Sciences, University of Michigan Medical School, Ann Arbor, MI, USA; 3 Division of Clinical Innovation, National Center for Advancing Translational Sciences, National Institutes of Health, Bethesda, MD, USA

**Keywords:** Implementation science, Learning Health Systems, real-world evidence, career development, translational science

## Abstract

Translation of research to practice is challenging. In addition to the scientific challenges, there are additional hurdles in navigating the rapidly changing US health care system. There is a need for innovative health interventions that can be adopted in “real-world” settings. Barriers to translation involve misaligned timing of research funding and health system decision-making, lack of research questions aligned with health system and community priorities, and limited incentives in academia for health system and community-based research. We describe new programs from the US Department of Veterans Affairs Health Services Research and Development (HSR&D) and the National Center for Advancing Translational Sciences (NCATS) Clinical and Translational Science Award (CTSA) Programs that are building capacity for Learning Health System research. These programs help to incentivize adopting and adapting Learning Health System principles to ensure that, primarily in implementation science within academic/veterans affairs health systems, there is alignment of the research with the health system and community needs. Both HSR&D and NCATS CTSA Program encourage researchers to develop problem-focused research innovations in partnership with health systems and communities to ultimately facilitate design treatments that are feasible in “real-world” practice.

## Introduction

The US health care system is complex and rapidly changing. These changes entail the needs and expectations of patients, as well as the increased demand on the clinicians, health systems, and communities who care for them. Ideally, research investments will also evolve to enable a timely and sustained public health impact. The rapid growth of new technologies, competition between health systems based on quality metrics and patient experience, and attention to community participation and engagement coincide with greater patient involvement in health care decisions, and subsequent increased demand for more rapid access to novel treatments. Research funding agencies may consider approaches that could broaden their focus beyond discovery and innovation to include supporting more rapid implementation of those discoveries in “real-world” practice.

In a landmark report on the Future of Health Services Research [1], the National Academy of Medicine (NAM) recommends that health care research initiatives: (1) promote more rapid and timely research that directly informs treatment implementation; (2) address priorities of health systems (i.e., problem-focused research); (3) involve patients, clinicians, their communities, and other stakeholders; and (4) build capacity within the scientific workforce that leads to more relevant research. These recommendations are also very relevant to the translational science spectrum [2] and are consistent with the Learning Health System framework [3,4]. Learning Health Systems [5] ensure continuous improvement of health outcomes through alignment of clinical informatics and organizational culture that promote scientific innovations that lead to the implementation of effective treatments [6].

Implementation science is a core component of Learning Health Systems [3] as it involves strategies to promote the more rapid uptake of effective treatments into “real-world” practice [7]. Increasingly, implementation science is becoming integral to the translational science spectrum [2,8]. A key reason for the growing interest in implementation science is the realization that many innovations in health care may never make it to routine clinical use because they are not designed for the changing needs of patients, their clinicians, or the health systems and communities that serve them [9].

Incorporating principles of implementation science in clinical and translational research mechanisms could help achieve Learning Health System goals and ensure that research findings are relevant to health systems and community stakeholders. Translational research has been defined as the field exploring the scientific and operational principles supporting the steps in the process [10]. The process is typically illustrated through four phases: T1 Research involves translation basic science advances to clinical interventions, including phase 1 clinical trials in highly controlled settings; T2 Research involves phase 2 and 3 clinical trials that inform clinical application and evidence-based guidelines; T3 Research refers to translation to wider practice, including comparative effectiveness research, health services, and implementation research; and T4 Research involves translation to communities, including sustained impacts on population-level outcomes and policy impacts [2].

Health systems, notably the Veterans Health Administration and Health Care Systems Research Network (e.g., Kaiser) [11], are seen as Learning Health Systems and, specifically, harbor implementation science (T3–T4) “laboratories” with the potential for embedding researchers who can study optimal approaches for scaling up and spreading treatment innovations that address health system priorities in routine practice more quickly [12]. Health systems are also essential environments for generating “real-world” evidence [13], primarily through the use of electronic health record data and pragmatic trial designs to test strategies for improving population health outcomes.

Nonetheless, there are limited research incentives to reward the rapid uptake of effective treatments and care models that address patient, provider, and health system priorities in “real-world” practice. Most researchers rely on investigator-initiated funding and peer-reviewed publications which remain the foundation for university promotions. There are few incentives and many disincentives for researchers to engage with health systems and communities to ensure the research fits with their priorities, to design studies that lead to implementation of treatments more quickly, or to do the hard work of partnering with stakeholders to get an innovation sustained in practice.

Increasingly, funding opportunities have arisen across federal funders that are focused on Learning Health System and implementation science. The Agency for Healthcare Research and Quality (AHRQ) and the Patient-Centered Outcomes Research Institute funded several institutions to support training in Learning Health System core competencies [14,15]. The National Heart, Lung, and Blood Institute (NHLBI) has also supported training opportunities in implementation science [16]. Other institutes such as the National Cancer Institute have funded centers and training resources devoted to implementation science [17]. Several requests for applications focused on implementation science and Learning Health Systems have emerged as well, notably through the National Institutes of Health (NIH) Helping to End Addiction Long-Term (HEAL) initiatives [18] and NHLBI [19–21].

This paper describes new approaches for implementation science that some agencies are taking to address barriers to translating discoveries to “real-world” practice based on Learning Health System goals. The focus is on the experiences of the US Department of Veterans Affairs (VA) Office of Research and Development (ORD) Health Services Research and Development (HSR&D) and the NIH National Center for Advancing Clinical and Translational Science (NCATS) Clinical and Translational Science Award (CTSA) Program. Both HSR&D and the CTSA Program support work that spans academic institutions and health systems to achieve “real-world” impact and move treatment discoveries to “real-world” implementation more quickly.

The principal audience for this paper is funding agencies supporting implementation science and research in Learning Health Systems in academic health systems (including VA affiliated hospitals). Academic health systems are the primary recipients of CTSA Program awards and VA funding that enable them to train and prepare the next generation of scientists. Researchers striving to build an academic career in Learning Health System and implementation research will also become familiar with these funding pathways as well. This paper does not involve human subjects, and the collaboration did not require review by an institutional review board (IRB).

## Barriers to Translation of Discoveries to “Real-World” Practice

Despite the increasing number of Learning Health System and implementation science research funding opportunities, some academic health systems may benefit from additional guidance in building research capacity in these areas. This is in part due to the inherent tension between innovation and application (implementation). Currently, most research mechanisms, especially those that focus on T1–T2 (pre-implementation biomedical and clinical research) do not specifically include methods that produce or adapt innovations that are relevant to “the health systems and communities they serve”. Moreover, some studies lack the capacity to test novel treatments or programs that cannot be taken up quickly in real-world practice.

To this end, we identified key barriers to translation of research into real-world practice that principally involve timing, framing, incentives, and capacity of competitively funded, investigator-initiated research projects, which comprise a good portion of research funding (Table 1). These key barriers were based on a recent conference on Embedded Research sponsored by the VA, AHRQ, and Kaiser [22] as well as recent literature focused on measuring impacts that serve to mitigate barriers toward building Learning Health Systems [12,23].

**Table 1.** Strategic investments to addressing common barriers to translation

Barrier	Programs and resources addressing barriers	
	NIH CTSA Program	VA HSR&D
*Timing*: Long timeline of traditional research funding mechanisms limits rapid development and implementation of new treatments, and lack of flexibility in changing health care landscape	Enterprise-wide informatics tools for health systems: for example, i-REX, SMART IRB, Accrual to Clinical Trials Center for Data to Health clinical care applications (EHR Dream Challenge, Maturity Model)	HSR&D Innovation Initiative: more rapid funding for high-risk projects that are responsive to health system needs QUERI programs: implementation strategy focused on deploying effective practices to achieve a national priority goal
*Framing*: Research questions have limited relevance and alignment with stakeholder priorities, and treatments lack feasibility for “real-world” practices to adopt (need for problem-focused research investments)	CEnR-Nav program Community Engagement Studios solicits perspectives about a scientific topic from community stakeholders Translational Research Studios: health system provider input on new discoveries or treatments for “real-world” use	COREs: access, suicide prevention, opioid/pain, virtual care Implementation plan requirement addressing stakeholders, strategy, and sustainment issues of investigator-initiated research project QUERI partnered evaluations: Mechanisms co-funded by VA operations leaders focused on rigorous implementation evaluation of national program or policy
*Incentives:* Researchers not rewarded to address health system issues or implementation into “real- world” settings	CTSA Program Collaborative Innovation Award promotes cross-cutting translational methods (e.g., implementation science) via U01 and R21 mechanisms University of Southern California-University of California at Los Angeles Implementation Science in LA County Program	Implementation Research Project mechanism (test implementation strategies in routine practice) QUERI partnered implementation initiatives addressing regional health system (VA Veterans Integrated Service Network) goals VA Researchers in Residence Program
*Capacity:* Limited training in research methods (e.g., informatics, complexity science, and implementation science) focused on provider and system factors to promote implementation of research findings into “real-world” settings	Development, Implementation, and Assessment of Novel Training in Domain-based Competencies (DIAMOND) initiative Institutional training awards, diversity supplement, diversity, and re-entry administrative supplements	HSR&D research priorities updated to include cross-cutting health services research methods: informatics/data science, complexity science/health systems engineering, and implementation science Career Development Awards and implementation science training (QUERI Center for Evaluation and Implementation Resources, NIMH Implementation Research Institute – VA sponsorship)

First, the timing of most research studies – 3 to 5 years for a typical investigator-initiated project funded by NIH or VA – means that as much as 6–8 years can elapse between when a question was conceived and when results are shared or published. Over that time, the health care landscape has changed and many of the contextual issues have shifted. Solutions that made sense when the research study was conceived may no longer be relevant or practical.

Second, researchers often struggle to frame research questions from the perspective of the stakeholders on whom uptake and implementation depends. Researchers frequently approach a health care problem as if the primary obstacle to progress is lack of evidence, when in reality the barrier is often a lack of ready pathways for implementation or problems of competing priorities and constrained resources in health systems or communities. For example, many treatments fail to be adopted by academic health systems because of a lack of an implementation plan or capacity to prepare and train existing clinicians in the use of the treatment once the research funding that supported treatment delivery goes away.

Third, current processes for funding and promotion do little to incentivize researchers who are dedicated to working on health system-level issues or implementing treatments or findings into practice. Current academic promotion pathways tend to reward grant funding and scientific publications, not the kind of work of building partnerships with health system leaders to develop pathways to sustain innovations. These activities may demand significant time without generating funding or publications. Where funding is available for implementation studies, they may require multiple sites to assess and intervene on provider and organizational factors affecting treatment uptake, while lacking routinely available data on patient and clinician outcomes to make them feasible.

Finally, the scientific field faces capacity problems – most training programs produce researchers who are skilled at analyzing data but may not be trained in how to study and change the behavior of individual clinicians and health systems. For example, VA research has benefitted greatly from collaborations from social scientists such as anthropologists but will still need better collaborations with system engineers to address complex operational problems facing health systems and provider behavior change that are part of a Learning Health System.

The VAʼs HSR&D program and the NCATSʼs CTSA Program have developed capacity building initiatives that overcome the disincentives of translational science from innovation to implementation in “real-world” health systems. In doing so, they build upon the VAʼs Research-to-“Real-World” Lifecycle [24] and the CTSA Dissemination and Implementation Science Workgroup findings [2], which outline how implementation science in particular can be applied to speed the deployment of research-to-“real-world” settings across the translational spectrum. As defined by NCATS and VA, implementation science involves the study of specific strategies (e.g., user-centered design, clinical tools, organizational methods, and public policies) derived from different scientific disciplines to promote the uptake of innovative treatments in “real-world” settings [7,8].

## NCATS CTSA Program

NIH awards grants to investigators at universities and other organizations through its 27 institutes and centers via a rigorous scientific review process. In 2006, Congress authorized the first of what is now called CTSA Program to provide funds to research teams at universities across the country to collaboratively identify solutions to common operational and scientific challenges observed in clinical and translational research. NCATS was established in 2011 and has supported the CTSA Program since then. It provides the continued support for this research ecosystem to promote all phases of clinical and translational science. Overall, the mission of NCATS is to “catalyze the generation of innovative methods and technologies that will enhance the development, testing, and implementation of diagnostics and therapeutics across a wide range of diseases and conditions” [25].

NCATS is built on the premise of enhancing implementation capacity for new treatments across the translation spectrum. NCATS supports over 50 national institutions, referred to as “Hubs,” which also identify other institutions in their community that can maximize the reach and impact of the Hubʼs work. The CTSA Program Hubs are designed to address challenges and opportunities to speed the translation spectrum in five major areas essential to a Learning Health System: informatics, common methods, interdisciplinary collaboration, community engagement and workforce development [26]. NCATS, through the CTSA Program, supports enterprise-wide standards for informatics tools to facilitate research collaborations (Table 1).

The CTSA Program has also supported novel strategies to ensure that community and patient perspectives are integrated throughout the development, testing, and implementation of scientific discoveries and potentially facilitate a treatmentʼs adoption and sustainability. The Community Engagement Studios solicits perspectives about a scientific topic from community stakeholders in order to promote a research pathway in its further study. Similarly, the Translational Research Studios are implemented by health care providers to elicit their perspectives on adopting new discoveries or treatments for “real-world” use [27]. The Community-Engaged Research Navigation (CEnR-Nav) program studies research questions generated from community partners [28].

## VA HSR&D Program

The VA HSR&D program is one of four research funding branches out of the US Department of Veterans Affairs ORD [12]. For over 90 years and with a current budget close to $800 million, ORD has funded VA-employed scientists to conduct research that is most relevant to Veterans. ORDʼs four branches of research funding span the translation spectrum, from basic (biomedical) to clinical science, rehabilitation research, and health services research (HSR&D). For the past 30 years, HSR&D has funded studies that examine and intervene on the organization, financing, management, and social factors of health care in order to improve the quality, cost, access, safety, and value of the health care delivered to Veterans.

HSR&D advances its goals through a combination of infrastructure support, career development, and research project funding. It supports 18 Centers of Innovation which address particular clinical priorities (e.g., pain, post-traumatic stress disorder, and womenʼs health) and support capacity building to advance the “basic science” of health services research methods including implementation science, complexity science (health systems engineering), and data (measurement) science. HSR&D also supports funding of competitive investigator-initiated projects and career development awards for early-stage investigators in these methods areas, with an eye toward Veteran-specific conditions (e.g., PTSD, traumatic brain injury, mental health conditions including suicide prevention, and substance use).

HSR&D is also one of the major “exporters” of implementation science in the USA notably though the Quality Enhancement Research Initiative (QUERI) program [29]. The mission of QUERI is to accelerate the adoption of research evidence into practice using implementation strategies, which are methods used to promote the uptake of interventions in “real-world” settings [8]. QUERI funds over 40 centers that are benchmarked on their ability to scale up and sustain effective practices especially in VA facilities with demonstrated quality gaps, and to rigorously evaluate the implementation process to inform national VA policy and practice investments.

HSR&D realized that to accelerate the translation of research into “real-world” settings it needed to change the way projects were solicited, reviewed, and funded. To address the long time frames of research projects and promote more attention to implementation and impact, HSR&D launched the Consortia of Research (CORE) initiative to build a national network of researchers and improve coordination, partnership, and priority setting with clinical/health system partners.

To address barriers related to the intervention to practice valley of death, HSR&D launched the Implementation Research Project (IRP) mechanism in 2019 to provide developmental funding for investigators to refine and pilot implementation strategies that support existing clinicians in the uptake of effective practices. IRPs focus on development and pilot-testing of specific implementation strategies in order to inform a fully powered hybrid effectiveness-implementation study. IRPs strive to close the gap between research and practice that is attributed to a lack of implementation strategies that enabled existing providers to be trained to deploy a treatment, rather than paying for providers on the research study, which was not sustainable once the research funding ended. Examples of implementation strategies developed through HSR&D and QUERI include Implementation Facilitation, Evidence-Based Quality Improvement, Audit and Feedback, and Design for Dissemination-Implementation [12].

Moreover, all HSR&D investigator-initiated funding mechanisms now require an implementation plan based on the QUERI Implementation Roadmap [8], as well as other strategies to promote Veteran and other stakeholder engagement. The implementation plan requires both a communication strategy to conveying results to health care leaders, providers, patients, and other stakeholders as well as an operational plan for applying the results of the study in “real-world” practice. Applications need to identify a health system operations leader who might potentially “own” the study results, and for treatment studies, the implementation of the intervention if proven effective. For Veteran/stakeholder engagement, applications need to provide details regarding how Veterans were engaged and the impact their input had on the study, how data on Veterans experience were ascertained, and how results will be disseminated to Veteran stakeholders.

Finally, to allow more rapid testing of higher risk health system innovations, HSR&D released an innovation initiative that provides 18 months of planning funds to see whether innovative, high-risk/high-impact ideas can be “de-risked” to point where it is worth investing larger amounts of funding (up to $500,000 per year for 4 years). Using a streamlined application and review process (three page applications, assessed on only two criteria: innovation and potential clinical impact), the initiative elicited a wide range of applications that proposed innovative tests of policy, data collaborations, or new partnerships to tackle five VA priority areas including suicide and opioid misuse.

## Discussion

Both the NCATS and VA incorporate principles of implementation science into their CTSA and HSR&D programs, respectively. Collaboration is a key characteristic in both programs. For example, the CTSA Program emphasizes community engagement, whereas HSR&D, with its research program embedded in a health care system, primarily has focused on involvement of health care providers and leaders. Table 2 provides examples of CTSA and VA programs that focus on Learning Health System capacity building [30]. Current efforts to measure impacts of these programs are underway [8,24].

**Table 2.** Examples of CTSA programs and VA-funded programs with Learning Health System initiatives

Program	Description	Goals and key impacts
Wakeforest College of Medicine CTSA Program	Wakeforest Clinical and Translational Science Institute	The CTSA Programʼs vision is to “be a catalyst for Wake Forest Baptist Healthʼs transition to a preeminent Academic LHS. (The) LHS focuses on the health of our community, maintains a strong pipeline of research findings and investigators across translational disciplines, and aligns science, informatics, incentives, and culture to continuously improve and innovate. (The LHS) embeds best practices seamlessly into the delivery process and capture new knowledge as a by-product of every interaction” https://ctsi.wakehealth.edu/About-CTSI
Vanderbilt University CTSA Program	Vanderbilt Institute for Clinical and Translational Research LHS program	“Bringing clinical research and clinical operations together as collaborative partners to … promote, develop, and support ventures aligned with the mission of LHS; ensure technical, procedural, and human infrastructure is in place for LHS evolution; and expedite efficient pursuit of activities for the purpose of improving the quality of patient care.” https://victr.vumc.org/our-programs/
VA HSR&D Consortium of Research (CORES)	Suicide Prevention Research Impact NeTwork	1) Facilitate help-seeking and engagement in care; 2) match level of risk to suicide prevention approaches, and tailor approaches to Veterans’ needs; and 3) implement, evaluate, and sustain evidence-based and promising interventions in a national network of investigators across US VA medical centers working in close collaboration with VA national leaders in mental health and suicide prevention
QUERI – VISN partnered implementation initiative	Consortium to Disseminate and Understand Implementation of Opioid Use Disorder Treatment (CONDUIT)	CONDUIT was a competitively awarded multi-regional quality enhancement program focused on a clinical priority chosen by VA VISN health system leaders which brings together implementation experts with health system leaders to scale up effective practices for opioid and pain treatment using implementation strategies. Success of CONDUIT is based on achievement of quality improvement goals (e.g., receipt of medication-assisted theory for opioid use disorder, receipt of guideline-concordant pain treatment) based on national clinical performance indicators.

VISN, Veterans Integrated Service Network.

Nonetheless, for communities and health systems to fully benefit from the research to real-world translation of discoveries, additional barriers will need to be overcome. Foremost is the limited capacity for Learning Health System and formal implementation science training. Recently, VA QUERI launched the Implementation Learning Hub program based on the QUERI Implementation Roadmap [8] to provide pathways for clinicians and clinical managers to learn how to deploy specific implementation strategies in their treatment setting. The CTSA program offers Diversity and Career Re-Entry Supplements to support a more diverse pool of translational scientists, and several CTSA Program awardees have also incorporated opportunities in implementation science which cover Learning Health System core competencies [14] such as engaging stakeholders in all aspects of the research process and transferring knowledge about implementation science to community practices [31]. Additional training programs that support researchers working across the translational “hinges” (e.g., from T1, T2, to T3 and T4) and facilitate the design of treatments that are feasible to implement in “real-world” health system settings could increase the number of young investigators prepared in to address these challenges.

Another barrier to larger clinical and implementation science trials is a lack of standardized protocols for data sharing and IRBs protocols that would facilitate collaboration between different health systems. The CTSA Program at the Medical College of Wisconsin and their local IRB office has developed an online “real-time” review process that has shortened the time from submission IRB review to final approval by 40% [32]. This innovation in infrastructure can aid Learning Health Systems implementing protocols more quickly than traditional review process.

In addition, researchers may benefit from additional funding opportunities that incentivize them to work closely with health system leaders to develop and implement effective innovations. Award mechanisms such as the CTSA Program Collaborative Innovation Awards, Limited Competition: Competitive Revision for CTSA Program^33^, and HSR&Dʼs Researchers in Residence programs can provide the funding stability for researchers to work directly with health system leaders and develop treatments with end users in mind. Research that positively impacts the health system and community outcomes including quality of care and sustainment of research innovations may potentially facilitate meaningful system-wide changes.

Through the experiences of the CTSA and VA HSR&D programs, there are key takeaways for academic health systems, researchers, and funders in promoting Learning Health System and implementation science research. First, academic health systems should encourage their researchers to take advantage of both capacity building and funding opportunities related to Learning Health System and implementation science through the CTSA Program and VA programs as well as other institutes and agencies (e.g., NHLBI and AHRQ). These avenues of funding can provide win–win collaborations with health systems leaders and promote real-world impacts on population health by enabling innovations to get into the hands of end users more quickly. Second, academic health systems should consider ways in which they can reward researchers that produce impacts on their overall quality of care, in addition to achieving traditional markers of academic productivity. Funders should in tandem monitor success based on impact metrics beyond publications and funding. Key impacts include improved quality of care (e.g., the HEALing Communities initiative is benchmarking grant recipients on reductions in opioid use mortality), whether innovations were spread beyond the original research study, and whether the research led to new technologies (invention disclosures) as well as changes in national programs or policies/legislation. These measures of impact are examples of metrics adapted from the NAM Degrees of Impact and specified in the VA Research Lifecycle framework [8].

Overall, strategic investments need to reflect Learning Health System principles and provide the appropriate incentives for academic health systems to support their investigators to collaborate with health system and community partners to test their innovations and overcome the chasm between treatment discovery and implementation. Novel initiatives through funding agencies such as VA and NIH followed by measures of real-world impacts will hopefully encourage researchers to develop innovations in partnership with health systems and communities and apply implementation science to get them tested and deployed more quickly to speed the translation from research findings to population health improvement to ultimately achieve “population health improvements.”

## References

[1] National Academy of Medicine. The future of health services research: Advancing health systems research and practice in the United States. National Academy of Medicine Press [Internet], 2018 [cited Nov 27, 2019]. (https://nam.edu/the-future-of-health-services-research-special-publication/) 37847808

[2] Leppin AM , et al. Situating dissemination and implementation sciences within and across the translational research spectrum. Journal of Clinical and Translational Science 2019; 1: 1–7. 10.1017/cts.2019.392 PMC7348034 32695482

[3] Guise JM , Savitz LA , Friedman CP. Mind the gap: putting evidence into practice in the era of learning health systems. JGIM 2018; 33(12): 2237–2239. 30155611 10.1007/s11606-018-4633-1 PMC6258636

[4] Friedman CP , Wong AK , Blumenthal D. Achieving a nationwide learning health system. Science Translational Medicine 2010; 2(57): 57cm29. 10.1126/scitranslmed.3001456 21068440

[5] Rubin JC , et al. Transforming the future of health together: the learning health systems consensus action plan. Learning Health Systems 2018; 2(3): e10055. 31245584 10.1002/lrh2.10055 PMC6508804

[6] Institute of Medicine: The Learning Healthcare System. National Academies Press [Internet], 2007 [cited Nov 27, 2019]. (https://www.nap.edu/catalog/11903/the-learning-healthcare-system-workshop-summary)

[7] Chambers DA , Feero WG , Khoury MJ. Convergence of implementation science, precision medicine, and the learning health care system: a new model for biomedical research. JAMA 2016; 315(18): 1941–1942. 27163980 10.1001/jama.2016.3867 PMC5624312

[8] Kilbourne AM , et al. Quality enhancement research initiative implementation roadmap: toward sustainability of evidence-based practices in a learning health system. Medical Care 2019; 57(Suppl 10 Suppl 3):S286–s293. 31517801 10.1097/MLR.0000000000001144 PMC6750196

[9] Kirchner JE , et al. Getting a clinical innovation into practice: An introduction to implementation strategies. Psychiatry Research 2019; 283: 112467. 31488332 10.1016/j.psychres.2019.06.042 PMC7239693

[10] Austin, CP. Translating translation. Nature Reviews Drug Discovery 2018; 17(7): 455–457. 10.1038/nrd.2018.27 PMC6023744 29674698

[11] Greene SM , Reid RJ , Larson EB. Implementing the learning health system: from concept to action. Annals of Internal Medicine 2012; 157(3): 207–10. 22868839 10.7326/0003-4819-157-3-201208070-00012

[12] Atkins D , Kilbourne AM , Shulkin D. Moving From discovery to system-wide change: the role of research in a learning health care system: experience from three decades of health systems research in the veterans health administration. Annual Review of Public Health 2017; 38: 467–487. 10.1146/annurev-publhealth-031816-044255 28125386

[13] Food and Drug Administration: Real-World Evidence. [Internet] [cited Nov 27, 2019]. (https://wwwfdagov/science-research/science-and-research-special-topics/real-world-evidence)

[14] Agency for Healthcare Research and Quality. Supporting the Next Generation of Learning Health Systems Researchers [Internet] [cited Feb 1, 2020]. (https://www.ahrq.gov/funding/training-grants/lhs-k12.html)

[15] Forrest CB , et al. Development of the learning health system researcher core competencies. Health Services Research 2018; 53(4): 2615–2632 28777456 10.1111/1475-6773.12751 PMC6051975

[16] National Heart Lung and Blood Institute. NHLBI K-12 grants will advance implementation science [Internet] [cited Feb 1, 2020]. (https://www.nhlbi.nih.gov/news/2017/building-workforce-translate-discoveries-health)

[17] National Cancer Institute Division of Cancer Control and Populations Sciences. Implementation Science [Internet] [cited Feb 1, 2020]. (https://cancercontrol.cancer.gov/IS/)

[18] NIH Helping to End Additional Long-Term (HEAL) Initiatives. [Internet] [cited Feb 1, 2020]. (https://heal.nih.gov/research/research-to-practice)

[19] National Heart Lung and Blood Institute. Preparing for Hybrid Effectiveness-Implementation Trials for Heart, Lung, Blood, and Sleep Diseases in the Inpatient Setting (U01) [Internet] [cited Feb 1, 2020]. (https://grants.nih.gov/grants/guide/rfa-files/RFA-HL-18-018.html)

[20] National Heart Lung and Blood Institute. Disparities Elimination through Coordinated Interventions to Prevent and Control Heart and Lung Disease Risk (DECIPHeR) and Stimulating T4 Implementation Research to Optimize Integration of Proven-effective Interventions for Heart, Lung, and Blood Diseases and Sleep Disorders into Practice (STIMULATE-2) [Internet] [cited Feb 1, 2020]. (https://grants.nih.gov/grants/guide/rfa-files/RFA-HL-20-003.html and https://grants.nih.gov/grants/guide/rfa-files/RFA-HL-21-011.html)

[21] National Institutes of Health. Dissemination and Implementation Research in Health [Internet] [cited Feb 1, 2020]. (https://cancercontrol.cancer.gov/IS/docs/DCCPS-DI-PAR-Factsheet.pdf)

[22] Damschroder L , et al. Embedded implementation research in health systems. Journal of General Internal Medicine, to appear.

[23] Braganza M , et al. The action framework for measuring impact in learning health systems. Journal of General Internal Medicine, to appear.

[24] Kilbourne AM , et al. Research lifecycle to increase the substantial real-world impact of research: accelerating innovations to application. MedicalCcare 2019; 57(Suppl 10 Suppl 3):S206–s212. 10.1097/MLR.0000000000001146 PMC6750195 31517789

[25] Collins FS. Reengineering translational science: the time is right. Science Translational Medicine 2011; 3(90): 90cm17. 10.1126/scitranslmed.3002747 PMC5101940 21734173

[26] National Center for Advancing Translational Sciences. [Internet] [cited Nov 27, 2019]. (https://ncats.nih.gov/ctsa/funding)

[27] Byrne DW , et al. Clinical and translational research studios: a multidisciplinary internal support program. Academic Medicine: Journal of the Association of American Medical Colleges 2012; 87(8): 1052–1059. 22722360 10.1097/ACM.0b013e31825d29d4 PMC3406254

[28] Kost RG , et al. Helping basic scientists engage with community partners to enrich and accelerate translational research. Academic medicine: Journal of the Association of American Medical Colleges 2017; 92(3): 374–379. 27119330 10.1097/ACM.0000000000001200 PMC5318154

[29] Kilbourne AM , et al. Accelerating research impact in a learning health care system: VA’s quality enhancement research initiative in the choice act Era. Medical Care 2017; 55(Suppl 7):S4–S12. 10.1097/MLR.0000000000000683 PMC5472006 27997456

[30] Atkins D. Are we growing the right health services research workforce of the future? thoughts from a national delivery system. Health Services Research 2018; 53(Suppl 2): 4034–4040. 30240010 10.1111/1475-6773.13032 PMC6149356

[31] Self WH , et al. Balanced crystalloids versus saline in noncritically ill adults. The New England Journal of Medicine 2018; 378: 819–828. 29485926 10.1056/NEJMoa1711586 PMC5846618

[32] Spellecy, R , et al. (2018). The real-time irb: a collaborative innovation to decrease IRB review time. Journal of Empirical Research on Human Research Ethics 13: 432–437. 29902956 10.1177/1556264618780803 PMC6146014

